# Clinical Distinctness of Allergic Rhinitis in Patients with Allergy to Molds

**DOI:** 10.1155/2016/3171594

**Published:** 2016-05-31

**Authors:** Krzysztof Kołodziejczyk, Andrzej Bozek

**Affiliations:** ^1^Allergy Outpatient Clinic ZWPS, Lompy 16, 40 038 Katowice, Poland; ^2^Clinical Department of Internal Medicine, Dermatology and Allergology, Medical University of Silesia in Katowice, MC. Sklodowskiej 10, 41-800 Zabrze, Poland

## Abstract

*Introduction*. Molds are a very diverse group of allergens. Exposure and sensitization to fungal allergens can promote the development and worsening of allergic rhinitis (AR).* Objective*. The natural course of allergic rhinitis was compared between a group of patients with allergy to molds and patients with AR to other allergens as the control groups.* Material and Methods*. The study group consisted of 229 patients, with a mean age of 27.4 ± 6.5 yrs. The study group was compared to groups of AR patients with allergy to house dust mites or pollens or with multivalent allergy. Allergic sensitization was assessed using the skin prick test (SPT) with a panel of 15 allergens to molds and other common inhalant allergens. Specific IgEs against all tested allergens were measured. Nasal fractional exhaled nitric oxide (FeNO) level was assessed with a chemiluminescence analyzer (NIOX MINO) and compared between groups. Cluster analysis was performed for determine models of AR in whole population.* Results*. Patients with allergy to mold have had AR with a higher blockage of nose than in the patients with other allergies.* Alternaria alternata* (59% of examined),* Cladosporium herbarum* (40%), and* Aspergillus fumigatus* (36%) were the predominant allergens in the study group. Patients with allergy to mold were more often present in two clusters: there were patients with more frequent accompanying asthma and high level of FeNO.* Conclusion*. Patients with allergy to molds have a significantly greater predisposition for bronchial asthma and high concentration of FeNO.

## 1. Introduction

The incidence of allergic rhinitis (AR) is increasing in most countries. AR affects approximately 500 million people and is a global health problem that affects the quality of life of persons in all age groups. AR is related to a wide variety of inhalant allergens, such as house dust mites, pollens, and molds [1, 2]. Diagnosing AR is often quite simple and is based on a typical history of nasal and nonnasal symptoms and the findings of diagnostic procedures, such as skin prick tests (SPT), specific IgE (sIgE) measurement, nasal provocation with allergens, cytology, and new techniques, including the nasal nitric oxide assay [1, 2]. However, there are differences in the clinical symptoms and seasonality of AR. Although many studies have analyzed the epidemiology, diagnostics, and treatment of AR caused by pollen and house dust mite allergy, information about AR with allergy to molds is still interesting and not entirely known [1, 3, 4].

*Objectives*. In the present study, a group of patients with allergy to molds and AR was analyzed and compared to a group of people with other allergies. The primary endpoint of the study was determining potential differences between the two groups.

## 2. Material and Methods

### 2.1. Patients

A total of 1750 patients (870 women and 880 men) between 18 and 86 years of age with chronic rhinitis treated at outpatient allergological clinics were prescreened. The preliminary diagnosis of AR was confirmed in 1450 patients on the basis of a retrospective history, positive skin prick tests (SPT), and measurement of the concentrations of serum specific IgEs to inhalant allergens. All patients were divided into four groups:

 With monovalent allergy to molds: group M.
 With monovalent allergy to pollens: group P.
 With monovalent allergy to house dust mites: group D.
 With multivalent allergy to two or more groups of allergens (house dust mites, molds, and pollens): group V.

The detailed characteristics of the patients are shown in Table 1.

### 2.2. Allergological Procedures

All analyzed patients underwent the following procedures.

(1) Skin prick tests (SPT) were performed with the following allergens:* D. pteronyssinus, D. farinae,* mixed grass, timothy-grass, rye, oat, wheat, corn, mixed tree, birch, alder, hazel, mugwort, nettle, and molds:* Aspergillus fumigatus, Cladosporium herbarum, Botrytis cinerea, Alternaria alternata, Curvularia lunata, Penicillium notatum, Fusarium moniliforme, Helminthosporium, Mucor mucedo, Trichophyton mentagrophytes, Rhizopus nigricans, Pullularia pullulans, Neurospora sitophila, Candida albicans,* and* Serpula lacrymans *(Allergopharma, Germany). Positive (histamine 10 mg/mL) and negative (saline) controls were also included. Allergy was defined as having a skin test positive for at least one allergen with a wheal max. diameter of at least 3 mm greater than that of the negative control. Patients with a negative test for histamine were excluded from further analyses.

(2) The response of serum immunoglobulin level (serum sIgE) to all mentioned allergens was measured using the immunoenzymatic test, Phadia Laboratory System (Phadia AB, Uppsala, Sweden). The results were assessed as positive when the serum sIgE concentration was >0.75 IU/mL (class II according to the manufacturer's brochure) [5].

(3) Careful examination of the eyes, ears, nose, throat, and nasal cytology was carried out for all patients. The severity of AR was assessed according to the Allergic Rhinitis and Its Impact on Asthma (ARIA) document. Patients with other nasal problems, such as chronic nasal obstruction, reduced sense of smell, bacterial infestation, and chronic sinusitis, were diagnosed on the basis of CT and nasal endoscopy. Because of these other nasal problems, some of those patients were excluded from further observations. Subjects with other clinically chronic or acute disorders and with a history of respiratory tract infection four weeks prior or during the study were also excluded.

(4) Allergic rhinitis symptoms were monitored using a 7-point visual graphic scale (published by the Joint Task Force and modified by ARIA) for grading the severity of nasal symptoms (sneezing, runny nose, congestion, itchy nose, and postnasal drip) and nonnasal symptoms (eye symptoms, throat symptoms, chronic cough, ear symptoms, headache, mental function, and quality of life). The symptoms were graded based on patient diaries who completed over one year of observation (every month over one year) [1].

(5) Measurements of the nasal fractional exhaled nitric oxide (FeNO) level were obtained using a hand-held chemiluminescence analyzer (NIOX MINO Airway Inflammation Monitor, Aerocrine AB, Solna, Sweden) calibrated with a nitric oxide calibration gas mixture. Nasal steroids, nasal decongestants, and antihistamine drugs were not applied within two weeks prior to the examinations. A single measurement was made both during nasal exhalation and after 10 seconds of breath-holding against an expiratory resistance of 5–25 cm H_2_O with a flow of 50 mL/s using a nasal mask. Measurements were performed during all months of one year. All measurements were repeated the following day, and all measurements were taken at the same time every month for one year. Three acceptable FeNO results and mean values were included in the analysis. The detection limit was 1 part per billion (ppb). The measurement range was 5–400 ppb.

(6) The* Alternaria* and* Cladosporium* spore counts were monitored for one year (2014) as typical seasonal allergens in South of Poland. Measurements concentration of the spores was carried out by using the apparatus volume Burkard:

- For* Alternaria*: there were 32 days (28 June–9 August) with the concentration above the threshold of 80 m^3^ of spores in the air (maximum 320 in 9 June).
- For* Cladosporium*: there were 21 days (25 June–12 August) with the concentration above the threshold of 2,800 m^3^ of spores in the air (maximum 5,800 in 15 June).

(7) The cluster analysis was performed to identify the different clusters (models) of AR associated (ARM) or not associated (ARNM) with molds allergy (as a positive SPT and/or sIgE to any mold allergens). *k*-means method was used. In the cluster analysis the following parameters were taken into consideration:

1. The severity of allergic rhinitis according ARIA [1] as follows:
  - “mild” AR means that none of the following items are present: sleep disturbance, impairment of daily activities, leisure and/or sport, impairment of school, or work symptoms present but not troublesome. These patients were excluded for further analysis.
  - “moderate/severe” AR means that one or more of the following items are present: sleep disturbance, impairment of daily activities, leisure and/or sport impairment of school, or work troublesome symptoms.
2. The presence of episodic or chronic bronchial asthma based on medical history and positive reversibility bronchial test.
3. The presence of pollen allergy including patients with clinical symptoms and a positive results of skin prick tests to grass, tree, or mugwort pollens and/or appropriate allergen specific IgE in serum.
4. The presence of house dust mites allergy and positive results of skin prick tests to* D. pteronyssinus* and* D. farinae* and/or appropriate allergen specific IgE in serum.
5. The presence of molds allergy as described above.

Serum concentration of total IgE and the mean nasal fractional exhaled nitric oxide levels (FeNO) from monthly measurements (as described above) were also used to cluster analysis. Total IgE and FeNO measures were log_10_-transformed to improve normality. Four-cluster models were tested and the proportion of ARM and ARNM were assessed for the different clusters.

### 2.3. Statistical Analysis

Data were analyzed with the statistical software package (STATISTICA ver. 8.1). Some parametric descriptive data were compared using Student's *t*-test. One-way analysis of variance (ANOVA) was used to compare the results of the allergy procedures between groups. The Mann-Whitney *U* test was used to compare FeNO values between groups, with *p* < 0.05. Spearman rang correlation test was used to estimate relation between counts spores and nasal symptoms.

The study was approved by the local ethics committee (Medical University of Silesia, Katowice, Poland).

## 3. Results

### 3.1. Skin Prick Tests and Specific IgE

In the group of patients monosensitized to molds, the prevalence of allergy to* Alternaria alternata* was approximately 59% (213–215 patients depending on whether they were positive by SPT and/or sIgE), that to* Cladosporium herbarum* was 40%, and that to* Aspergillus fumigatus* was 35%. The results of the SPT and sIgE measurements are shown in Table 2.

### 3.2. Clinical Features of Allergic Rhinitis

Patients monosensitized to molds more frequently have had an intermittent AR compared to the other groups (this was also true for the patients with pollen allergy). The detailed results are presented in Table 3. Patients with allergy to* Alternaria alternata* or* Cladosporium herbarum *had a significantly higher risk of intermittent AR as follows: hazard ratio (HR) = 1.62 (95% CI: 1.12–1.77) and HR = 1.83 (1.73–1.95). Furthermore, patients with allergy to* Aspergillus fumigatus* and* Penicillium notatum* had a significantly higher risk of chronic AR as follows: HR = 1.79 (1.85–1.99) and HR = 1.48 (1.32–1.75), respectively. No such correlations were observed for other species of molds. Patients with mold allergy were significantly more frequently diagnosed with chronic sinusitis than the patients in the other groups as follows: in group M, 49 patients (20.5%)^*∗*^; in group D, 45 patients (11.8%); in group P, 22 patients (6.0%); and in group V, 46 patients (10.8%) (ANOVA, ^*∗*^ *p* < 0.05).

The mean daily symptoms score (the monthly mean value of the daily total scores for nasal and nonnasal symptoms) revealed the following:

(i) Congestion and itchy nose were observed significantly more frequently in patients with allergy to molds than in patients with allergy to pollen or to multiple allergies (group V) (*p* = 0.005).
(ii) Sneezing and runny nose dominated in patients with pollen allergy and multivalent allergy (*p* = 0.004).

There were no significant differences in the types of nasal symptoms between the patients with allergy to molds and the patients with allergy to house dust mites (*p* = 0.21).

There were positive correlations between the mean daily nasal symptoms score and concentration of spores in the air during pollen season for* Alternaria* (Spearman rank correlation test *r* = 0.77, *p* = 0.005) and* Cladosporium* (*r* = 0.81, *p* = 0.003).

Patients with allergies to molds were more frequently diagnosed with bronchial asthma than patients in the other groups as follows: in group M, 138 patients (57.7%)^*∗*^; in group D, 132 patients (34.7%); in group P, 115 patients (31.4%); and in group V, 187 patients (44%) (ANOVA, ^*∗*^ *p* < 0.05). There was a particularly high risk of asthma in patients who were sensitized to* Aspergillus fumigatus* or* Alternaria alternata*: HR = 1.31 (1.18–1.42) and HR = 1.42 (1.38–1.5), respectively.

### 3.3. The FeNO Levels (Mean of 12 Measurements per One Year)

The test-retest reliability with the intraclass correlation coefficient (ICC) of FeNO was 0.82 (range 0.73–0.91). The mean value of FeNO was 48.1 ± 12.9 ppb for all study population.

Patients in group M had significantly higher mean one-year FeNO levels than patients in the control groups (D, P, and V): 67 ± 11.5 ppb^*∗*^ (group M), 45.2 ± 9.1 ppb (group D), 51.4 ± 10.9 (group P), and 56.1 ± 11.6 (group V) (^*∗*^ANOVA test for *p* < 0.05).

There was a statistical lower FeNO level in group P than in group M during the pollen season (May–August): 46.9 ± 8.5 ppb versus 78.011 ± 16.6 ppb, respectively (*p* < 0.05).

Patients with monovalent allergy to* Alternaria* and/or* Cladosporium* have had increased mean FeNO from 34.78 ± 26.1 ppb during winter months (December–March) to 69.11 ± 30.9 ppb during season: from June to August (*p* < 0.05).

### 3.4. Cluster Analysis

**Figure 1:**
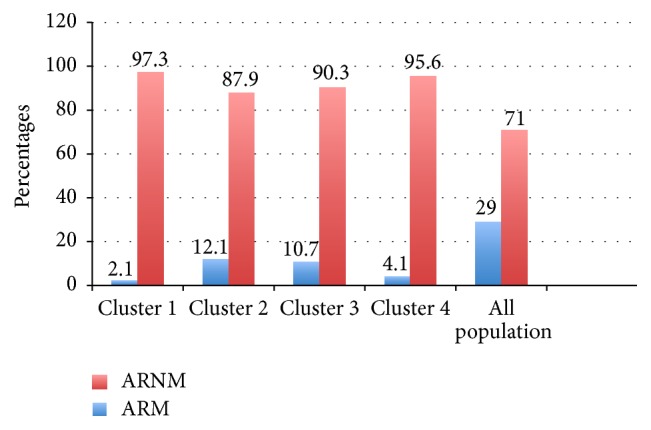
Proportion of patients with AR with (ARM) or without (ARNM) molds allergy for each cluster (1–4).

A four-cluster model was determined (Table 4). Patients with ARM were especially concentrated in clusters 2 and 3 (Figure 1). There were higher concentration of FeNO higher predisposition to asthma and less concentration of patients with severe AR than in clusters 1 and 4.

## 4. Discussion

The results revealed the great variety in the course of AR in patients with allergy to molds, from intermittent to chronic and from mild to severe AR. The course is largely dependent on the type of allergen sensitization. This is confirmed by data from the literature [6–8]. In this study population, the most common sensitization was to* Alternaria* and* Cladosporium*, and this finding is widespread and has been confirmed by other studies [1, 6, 7]. A few trends were found in the patients who were monosensitized to molds that were not present in other allergen groups. First, in the patients monosensitized to molds, the symptoms of AR were mainly blockade and itchy nose, which were different from the predominate symptoms in patients with AR due to other allergens. These symptoms often resemble nonallergic rhinitis, especially in patients with a negative common inhalant allergen SPT and without extensive testing of allergies to molds. This may cause these patients to be incorrectly diagnosed and cause problems with their treatment. We observed this phenomenon in approximately 15% of our patients. No similar information was available in the literature, and further studies are needed.

Second, and of great importance, patients who are monosensitized to molds suffer significantly more frequently from chronic sinusitis and bronchial asthma. The high prevalence of chronic rhinosinusitis in these patients is consistent with the findings of previous studies [1, 6]. However, in patients with chronic rhinitis with polyps, there was no correlation between the disease and allergy (including molds), but fungus was present in the sinuses [9]. A limitation of our study is that only the IgEs related to mold allergy were examined.

The high risk of asthma observed in patients who were monosensitized to molds was in agreement with the findings of other studies [6, 8, 10]. Longitudinal studies have shown increased exposure to indoor fungi before the development of asthma symptoms, which suggests that some species pose a respiratory health risk in susceptible populations or can exacerbate current asthma [10]. The greater risks of asthma in patients who were allergic to* Alternaria, Cladosporium,* and* Aspergillus* are consistent with previous observations [6, 10]. However, it seems that the overall dominance of allergy to those species determined this dependence.

Our publication was not focused on asthma patients; however, that will be a subject of future research.

Nasal nitric oxide is a good marker of inflammatory diseases of the upper airways, such as allergic rhinitis [11–13]. There are two methods to assess upper airway NO level. Nasal NO is measured as air flows through the nasal cavities: air is aspirated or insufflated with a target airflow rate through one nostril while the velum is closed during breath hold. Measurement of nasal fractional exhaled NO (nasal FeNO) is the second method: the patient exhales nasally through a tight facemask with a fixed flow. Nasal FeNO represents only a fraction of endogenous NO in contaminated air passing through the nose at a high flow rate. These two methods are recommended by ATS [14]. Nasal FeNO levels are lower and more variable than nasal NO levels. The FeNO measurements were validated using a hand-held analyzer in previous studies [9, 15]. The elevation of nasal FeNO level in patients with allergy to molds compared to that of patients in the other groups suggests more active inflammation in the upper airways in patients with allergy to molds. This may explain the higher incidence of bronchial asthma in patients allergic to molds.

The significant changes of FeNO level during the pollen season were particularly evident in patients with allergy to pollens and outdoor molds (*Alternaria* and* Cladosporium*). The nasal NO level was slightly elevated during the season when the air concentrations of grass pollen and Alternaria spores were very high. However, during and after the pollen season, there was no significant correlation between nasal symptom scores and nasal FeNO in any of the allergic patients. The value of FeNO did not correlate with any type of allergen.

It is worth emphasizing that patients with allergic rhinitis and allergy to mold focused on two models as a result of cluster analysis of all study population. Patients with accompanying asthma and high concentrations of FeNo were followed in these clusters. This further confirms the distinctiveness of patients with AR and allergy to molds.

## 5. Conclusion

Patients with allergy to molds have a clinically milder type of AR; however, they have a significantly greater predisposition for bronchial asthma. The value of FeNO did not correlate with any type of allergen, but it was significantly higher in patients with monovalent allergy to molds.

## Figures and Tables

**Table 1:** Characteristics of study groups.

Features	Total analyzed patients *n* = 1450			
	Group M *n* = 239	Group D *n* = 380	Group P *n* = 366	Group V *n* = 425
Duration of allergy in years (±SD)	9.0 ± 4.2^*∗*^	6.4 ± 1.3	5.2 ± 7.2	6.1 ± 2.9
Residence: the village	24.4%	19.7%	22.1%	20.3%
Higher education	22.7%	24.5%	23.2%	19.9%
Allergy in the family	37.5%	34.5%	41.1%	40.1%
Allergy in childhood	32.1%	39.9%	28.7%	55.3%^*∗*^
Total serum IgE U/L (mean ± SD)	79.4 ± 14.6^*∗*^	133.4 ± 65.2	145.3 ± 78.2	155.6 ± 92.0

SD: standard deviation, group M: monovalent allergy to molds, group D: monovalent allergy to house dust mites, group P: monovalent allergy to pollens, and group V: multivalent allergy; ^*∗*^significant differences between groups, *p* < 0.05.

**Table 2:** The mold allergy profile of the studied patients.

Species of fungi	Patients with mold allergy *n* = 239 (group A)	
	Positive PTS result, *n* (%)	Increased concentration of sIgE, *n* (%)
*Aspergillus fumigatus*	85 (35.6)	86 (36.0)
*Cladosporium herbarum*	96 (40.2)	97 (40.6)
*Botrytis cinerea*	25 (10.5)	25 (10.5)
*Alternaria alternata*	215 (57.7)	213 (89.1)
*Curvularia lunata*	22 (9.2)	23 (9.6)
*Penicillium notatum*	14 (5.9)	14 (5.9)
*Fusarium moniliforme*	53 (22.2)	54 (22.6)
*Helminthosporium*	45 (18.4)	45 (18.4)
*Mucor mucedo*	75 (31.4)	75 (31.4)
*Trichophyton mentagrophytes*	66 (27.6)	67 (28.0)
*Rhizopus nigricans*	40 (16.7)	40 (16.7)
*Pullularia pullulans*	15 (6.3)	15 (6.3)
*Neurospora sitophila*	22 (9.2)	22 (9.2)
*Candida albicans*	35 (14.6)	32 (13.4)
*Serpula lacrymans*	40 (16.7)	40 (16.7)

PTS: skin prick tests and sIgE: specific IgE.

**Table 3:** Characteristics of allergic rhinitis in study groups according to ARIA.

AR	Total analyzed patients *n* = 1450			
	Group M *n* = 239 (%)	Group D *n* = 380 (%)	Group P *n* = 366 (%)	Group V *n* = 425 (%)
Intermittent	78 (32.7%)^*∗*^	55 (14.5%)	315 (86.1%)^*∗*^	112 (26.4%)
Chronic	123 (51.5%)	254 (66.8%)	41 (11.2%)^*∗*^	301 (70.8%)
Mild	65 (27.2%)^*∗*^	54 (14.2%)	71 (19.4%)	58 (13.7%)
Moderate	88 (36.8%)^*∗*^	191 (50.3%)	209 (57.1%)	213 (51.1%)
Severe	48 (20.1%)	64 (16.6%)	76 (20.8%)	142 (33.4%)^*∗*^

^*∗*^Significant difference compared with other groups analyzed (ANOVA test), *p* < 0.05.

**Table 4:** Cluster analysis from whole population (*n* = 1450).

*n* = 1450	Cluster 1 *n* = 385	Cluster 2 *n* = 431	Cluster 3 *n* = 243	Cluster 4 *n* = 391
Severity^∧^				
(i) Moderate	23.8	25.3	31.2	49.4
(ii) Severe	16.8	9.1	8.2	23.1
Asthma^∧^	11.2	32.1	28.3	5.8
Pollen allergy^∧^	78.5	36.3	11.3	87.4
House dust mite allergy^∧^	18.8	33.5	32.1	51.5
Total IgE^*∗*^	138 (129)	89 (87)	116 (98)	221 (167)
FeNO^*∗∗*^	35 (22)	69 (54)	54 (34)	36 (15)

^∧^The proportion of variables in each cluster were shown as percent. ^*∗*^log_10_⁡ total IgE (SDs) and ^*∗∗*^log_10_⁡ mean FeNO (SDs).
